# Development and application of an indirect ELISA for the detection of antibodies to porcine epidemic diarrhea virus based on a recombinant spike protein

**DOI:** 10.1186/s12917-018-1570-5

**Published:** 2018-08-20

**Authors:** Huixing Lin, Hong Zhou, Lu Gao, Bin Li, Kongwang He, Hongjie Fan

**Affiliations:** 1 MOE Joint International Research Laboratory of Animal Health and Food Safety, College of Veterinary Medicine, Nanjing Agricultural University, Nanjing, 210095 China; 2 Institute of Veterinary Medicine, Jiangsu Academy of Agricultural Sciences, Nanjing, 210014 China; 3 Jiangsu Co-Innovation Center for the Prevention and Control of Important Animal Infectious Diseases and Zoonoses, Yangzhou University, Yangzhou, 225009 China

**Keywords:** Porcine epidemic diarrhea virus, Spike protein, ELISA

## Abstract

**Background:**

As the major causative agent of swine viral diarrhea, porcine epidemic diarrhea virus (PEDV) has caused massive losses to the economies of swine raising countries. Accordingly, the serological detection of corresponding antibodies would be beneficial to diagnose PEDV indirectly to control the disease. In this study, an indirect enzyme-linked immunosorbent assay (ELISA) based on the recombinant truncated spike (S) protein of PEDV was developed and validated.

**Results:**

The reaction conditions of the developed indirect ELISA were optimized. This indirect ELISA was compared to indirect immunoinfluscent assay (IFA), and the overall coincidence rate was 96.74% based on testing 368 clinical serum samples with different PEDV antibody levels. No cross-reactivity with other common swine pathogens was detected for the developed S1 indirect ELISA. Finally, the S1 indirect ELISA was applied to detect serum antibodies of 3304 field samples collected from different pig farms in eastern China, and it presented an overall substantial agreement on the PEDV infection status.

**Conclusions:**

This established S1 indirect ELISA is capable of detecting serum antibodies against PEDV, and due to its high sensitivity and specificity, it could be applied for serological evaluation and indirect diagnosis of PEDV infection.

## Background

Porcine epidemic diarrhea (PED) is a highly contagious swine enteritis caused by porcine epidemic diarrhea virus (PEDV), which belongs to the order *Nidovirales* and family *Coronaviridae*. The typical symptoms of PED are diarrhea, vomiting, and dehydration, which can be especially dangerous to suckling piglets [1, 2]. The mortality of neonatal piglets younger than 5 days old can approach 100% [3–5]. PED first appeared in Britain in 1971, followed by an outbreak of diarrhea in several pig farms in Belgium in 1977 [6]. These outbreaks led to identification of a coronavirus-like particle named CV777, which is now recognized as the classic PEDV strain. In recent years, PED epidemics have become prevalent in swine-raising countries in Asia, including South Korea, China, Japan, and Vietnam, and can cause enormous economic loss [7, 8].

PEDV is a single-stranded RNA virus composed primarily of four structural proteins: the spike protein (S, 180–220 kDa), membrane protein (M, 27–32 kDa), envelope protein (E, 7 kDa) and nucleocapsid protein (N, 55–58 kDa). S protein is located on the surface of the virus particle. It is categorized as a type I membrane fusion protein and has the significant biological effect of binding to target cell receptors and entering the cell through plasma membrane fusion [9, 10]. The S protein has higher antigenicity than any of the other PEDV proteins, and anti-S antibodies detected in PEDV-infected pigs persist longer than anti-N antibodies [11]. The S protein can be separated into the S1 (1–789 aa) subunit and the S2 (790–1383 aa) subunit [12]. The S1 subunit is the extracellular domain and can recognize and bind to target cell receptors [13], and it is closely linked to the formation of neutralizing antibodies. Therefore, this study selected a gene fragment within the S1 subunit as a coating antigen to develop an indirect enzyme-linked immunosorbent assay (ELISA) method for the detection of PEDV antibodies.

## Methods

### Materials

The PEDV YC2014 strain was isolated on a breeding farm in Yancheng city in 2014 (GenBank: KU252649.1). The prokaryotic expression vector pET-28a(+) was purchased from BioVector NTCC Inc. (Beijing, China). The HRP-goat anti-pig IgA, HRP-goat anti-pig IgG, and FITC-goat anti-pig IgA was purchased from Abcam plc. (Shanghai, China). The standard PEDV negative serum were collected from specific pathogen free (SPF) pigs. The standard PEDV positive serum were collected from experimentally PEDV immunized SPF pigs, at 7, 14, 21, 28, 35, 42 and 49 day post-inoculation (dpi). These standard serum were identified of PEDV-specific antibodies positive by both indirect immunoinfluscent assay (IFA) and seroneutralization assay (SN) as previously described [14, 15]. The swine PoRV antibody ELISA kit and the swine TGEV ELISA kit were obtained from Ingenasa (Madrid, Spain).

### Construction of the recombinant plasmid

The gene sequence of truncated spike protein (named *S1*) was amplified from the genomic RNA of PEDV YC2014 strain (Genbank: KU252649.1) by reverse-transcriptase (RT)-PCR. The forward primer was 5’ CGCGGATCCGTCACTAGGTGCCAGTCCACTATTAA-3’and the reverse primer was 5’-CCCAAGCTTTCAATTGTAAATATCCACTTTAAGAAAACAATAA-3′. Underlined portions represent *Bam*H I and *Hin*d III restriction sites, respectively. The target gene was 1068 bp in length and subcloned into the prokaryotic expression vector pET-28a(+), then transformed into a strain of competent *E. coli* cells, DH5α. Transformed colonies were selected from Luria-Bertani (LB) agar plates containing kanamycin (50 μg/mL) and were identified by PCR. The resulting recombinant expression plasmid was named 28a-S1 and was identified by double enzyme digestion and DNA sequence analysis. Subsequently, the recombinant expression plasmid 28a-S1 was transformed into *E. coli* BL21 (DE3).

### Expression and purification of the recombinant protein S1

The positive transformants were cultured in LB medium containing 50 μg/mL kanamycin with vigorous shaking at 37 °C until the 600 nm optical density (OD_600_) of bacteria cultures reached approximately 0.5. Then, by adding isopropyl-β-D-thiogalactopyranoside (IPTG), the recombinant protein S1 was induced for 6–8 h at 37 °C. The expression of recombinant proteins was analyzed by 12% (*v*/v) sodium dodecyl sulfate-polyacrylamide gel electrophoresis (SDS-PAGE) and the gels were stained with coomassie brilliant blue. Induced cells were pelleted and washed in phosphate-buffered saline (PBS) three times, then lysed by sonication in an ice-water bath. The lysed cells were centrifuged at 12,000 g for 10 min, then the precipitate (inclusion body) was dissolved with binding buffer containing 8 M urea. The supernatant and the precipitate were then subjected to SDS-PAGE analysis. The recombinant protein was purified through affinity chromatography using a Ni-NTA spin column following the manufacturer’s recommendations.

### Western blotting

Purified recombinant protein S1 was subjected to 12% (*v*/v) SDS-PAGE, and the gel was prepared for western blotting as follows. Recombinant proteins separated in the gel were electrically transferred to a nitrocellulose membrane and the membrane was blocked overnight at 4 °C with Tris-buffered saline containing Tween-20 (TBST) which contained 5% (*w*/*v*) skimmed milk powder. The composition of TBST is as follows: 20 mM Tris-HCl, 150 mM NaCl, and 0.05% Tween-20. The membrane was then incubated with pig anti-PEDV polyclonal antibody (1:300 dilution in blocking buffer) for 1 h at 37 °C on a plate shaker. Following this incubation, the membrane was washed three times with TBST buffer and reacted with HRP-goat anti-pig IgG (1:2000 dilution in TBST) at 37 °C for 45 min. After three washes, the final color reaction was developed with a solution of 3,3′-Diaminobenzidine (DAB).

### Recombinant protein S1 indirect ELISA

Conventional indirect ELISA was performed with the following steps. ELISA plates with 96 wells (Costar, USA) were coated with 100 μL purified recombinant S1 protein in bicarbonate buffer (pH = 9.6) for 2 h at 37 °C. Then, plates were washed three times with PBST (PBS containing 0.05% Tween-20) and blocked with 5% skimmed milk in PBS for 2 h at 37 °C. After plates were washed, 100 μL porcine serum samples diluted in PBS containing 5% (*w*/*v*) skimmed milk was added and incubated for 45 min at 37 °C. Plates were washed four times and reacted with 100 μL diluted secondary antibody (HRP-goat anti-pig IgA or HRP-goat anti-pig IgG) for 30 min at 37 °C for the purpose of detecting IgA or IgG against PEDV in serum samples. Plates were then washed four times and 100 μL tetramethylbenzidine (TMB) substrate solution was added to each well for a chromogenic reaction at room temperature for 15 min in complete darkness. The color reaction was stopped by the addition 50 μL of 2 M H_2_SO_4_ to each well. Finally, the OD_450_ was measured and recorded immediately using an Infinite 200 PRO microplatereader (Tecan, Männedorf, Switzerland).

The optimal dilution of recombinant protein S1 and standard serum was determined by a checkerboard titration based on the method mentioned above. Briefly, the concentration of S1 protein was gradually reduced in the following series: 10, 7.5, 5, 2.5, 1.0, and 0.5 μg/mL. The standard PEDV positive and negative serum were serially diluted in a 2-fold series from 1:20 to 1:320. When the OD_450_ ratio of positive serum to negative serum was highest, and the OD_450_ of positive serum was closest to 1.0, the corresponding dilutions of coated antigen and serum sample were considered optimal. In addition to optimal protein dilution, the coating conditions, blocking solution, and reaction time of various materials was explored. Furthermore, the optimal concentration of HRP-goat anti-pig IgA was tested using the following dilutions: 1:2000, 1:5000, 1:10000, 1:15000, and 1:20000.

### Determination of the cut-off value

Two hundred and seventy serum samples were collected for the purpose of determining the positive-negative cut-off value, of which 90 serum samples were collected from 90 SPF pigs, 180 serum samples were collected from grow-finish pigs from five farms between 2009 and 2012. These five farms were located in areas with no previous history of enteric signs compatible with viral diarrhea, and were PEDV RNA negative by real-time RT-PCR based on a single collection. These serum samples were confirmed PEDV-negative by both IFA and SN assays as previously described [14, 15], and then were used to define the cut-off value in the S1 indirect ELISA. The OD_450_ value of these PEDV-negative serum samples obtained in this S1 indirect ELISA were recorded to calculate the cut-off value. The mean OD_450_ value of these negative samples (N) + 3 × standard deviations (SD) was defined as the cut-off value. Serum samples showing OD_450_ value greater than or equal to this cut-off were considered PEDV-seropositive.

### Assessment of S1 indirect ELISA

To assess the accuracy of this developed S1 indirect ELISA, 368 serum samples from different pig farms were tested using this ELISA method. As a comparison, IFA was applied to test these samples and act as a reference method to distinguish positive or negative samples. Briefly, Vero cells grown on 96-well plates were infected with the PEDV YC2014 strain at multiplicity of infection (m. o. i) of 5. At 48 h post-infection, cells were washed three times with PBST and fixed with cold methanol for 10 min at − 20 °C. Cells were then washed three times with PBST and blocked with 10% bovine serum albumin (BSA) at 37 °C for 1 h. After been double diluted for six consecutive dilutions in dilution buffer (1% BSA in PBST), the 368 serum samples with varied PEDV antibody status were added in the wells of 96-well plate, and were incubated for 1 h at 37 °C. After three washes with PBST, cells were treated with a FITC-conjugated goat anti-pig IgA (Thermo Scientific) at a 1:500 dilution with PBS for 30 min at 37 °C. After a final four washes with PBST, all wells were examined using fluorescence microscopy (Axio Observer Z1, Zeiss, Germany). The PEDV antibody titers of the serum samples were expressed as the highest dilution of serum samples producing green fluorescent in the wells of 96-well plates.

The results of this two methods were compared, and the sensitivity and specificity of detection were calculated to evaluate the accuracy of the S1 indirect ELISA. Sensitivity was defined as the ratio of positive tests from the developed S1 indirect ELISA to the positive tests from the IFA. Specificity was defined as the ratio of negative tests from the developed ELISA to the negative tests from the reference IFA.

### Serum cross-reactivity of S1 indirect ELISA to other pathogens

To validate the cross-reactivity, this S1 indirect ELISA was utilized to test porcine serum positive for other swine pathogens, namely, porcine transmissible gastroenteritis virus (TGEV), swine rotavirus (PoRV), porcine kobuvirus (PKV), porcine bocavirus (PBoV), porcine norovirus (PNoV), porcine circovirus type 2 (PCV2), porcine reproductive and respiratory syndrome virus (PRRSV), and enterotoxigenic *E. coli* (ETEC), *Jerson Prand* of the small intestine, *Clostridium Welchii* Type C. The positive sera were prepared by our lab, by immunizing the specific pathogen free (SPF) piglets with purified virus or bacteria. Thirty positive serum samples for each virus were tested, and each sample was repeated in triplicate.

### Determination of repeatability of S1 indirect ELISA

To test the repeatability of this ELISA, 255 serum samples with different PEDV antibody levels were chosen. For inter-assay variability, each sample was tested in 5 replicates on plates of different occasions. For intra-assay variability, each sample was tested in 5 replicates on plates within the same occasion. The results were presented as the coefficient of variation (CV), which is the ratio of the standard deviation (SD) to the mean OD_450_ value of each group of samples (S). A CV value criterion of 10% was used to meet the repeatability requirement of the test.

### PEDV antibody detection of field serum samples

This S1 indirect ELISA was applied to seroepidemiological analysis of a total of 3304 clinical swine serum samples collected from thirty seven farms in eastern China. The PEDV infection status of a given farm was determined based on demonstration of PEDV RNA in fecal samples by real-time RT-PCR and presence of enteric signs [16]. One thousand one hundred and twenty five serum samples were collected from nursery and grow-finish pigs from ten farms between 2011 and 2015 at 3–6 weeks after the start of PEDV outbreaks in these farms. Four hundred and eighty two serum samples were collected from nursery and grow-finish pigs from five farms between 2012 and 2015. These five farms were located in areas with no previous history of enteric signs compatible with viral diarrhea, were PEDV RNA negative by real-time RT-PCR based on a single collection, and were considered non-exposed to PEDV. Three hundred and forty four serum samples from nursery pigs without PEDV exposure were collected from six farms between 2011 and 2015. These samples were confirmed to be positive for anti-PoRV antibodies (3 farms, *n* = 149) or anti-TGEV antibodies (3 farms, *n* = 195) by both IFA and commercial ELISA kits (obtained from Ingenasa). One thousand three hundred and fifty three porcine serum samples with unknown PEDV exposure status were randomly selected from sixteen farms from nursery and grow-finish pigs between 2011 and 2014.

## Results

### Subcloning, inducible expression and purification of recombinant S1 protein

**Fig. 1:**
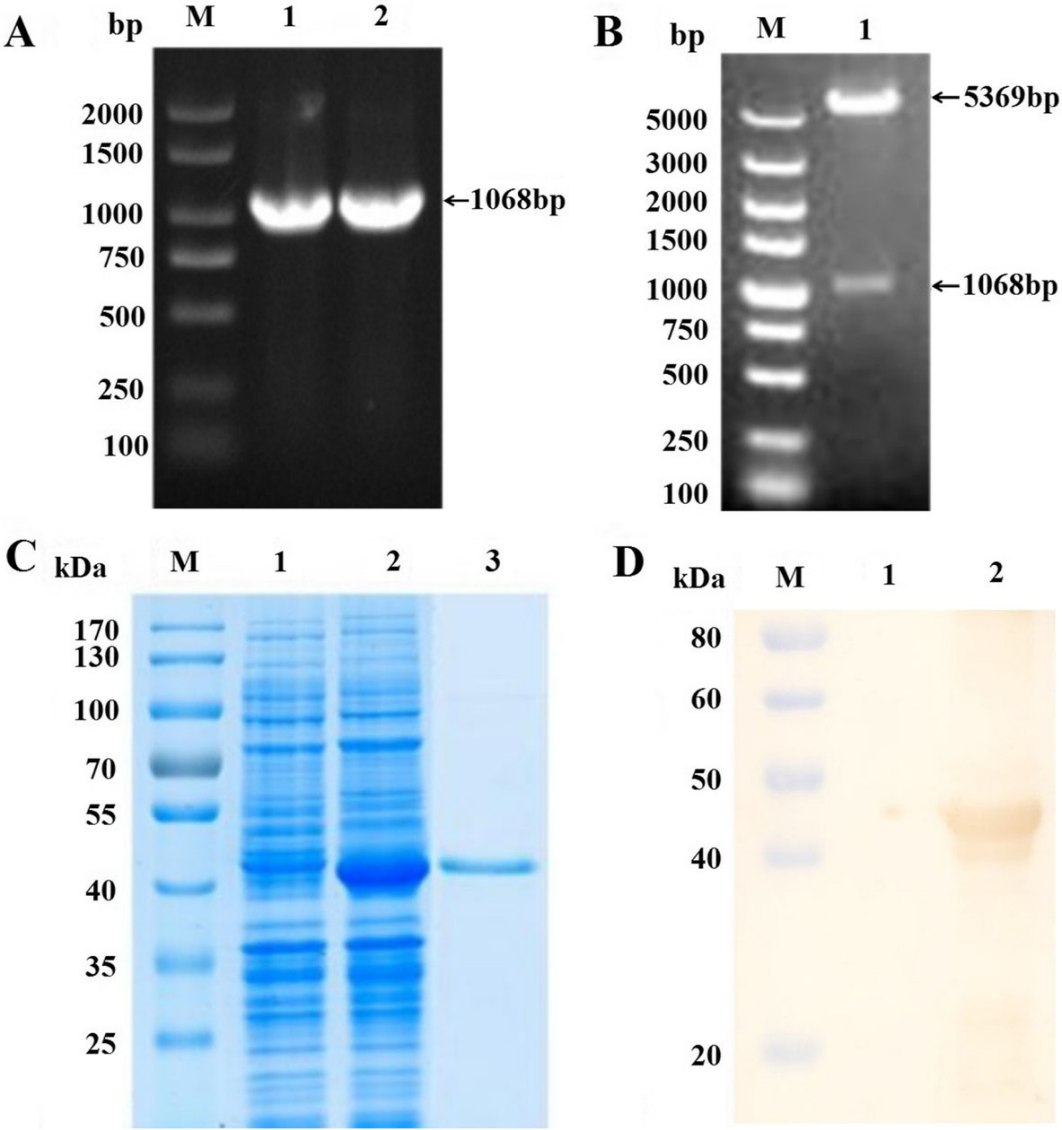
Amplification, SDS-PAGE and western blotting analysis of the recombinant protein S1. **a** RT-PCR amplification of the truncated S1 gene fragment. Lane M, DL2000 DNA marker. Lane 1 and 2, the truncated S1 gene fragment. **b** Identifiction of the recombinant expression plasmid 28a-S1 by double enzyme digestion. Lane M, DL5000 DNA marker. Lane 1, the recombinant expression plasmid 28a-S1 digested by *Bam*H I/*Sal* I. **c** SDS-PAGE analysis of S1 protein. Lane M, prestained protein molecular weight standard. Lane 1, transformed cells of BL21/pET-28a(+) after IPTG induction for 6 h. Lane 2, transformed cells of BL21/28a-S1 after IPTG induction for 6 h. Lane 3, purified recombinant protein S1 by affinity chromatography of Ni-NTA spin column. **d** Western blotting analysis of S1 protein. Lane M, prestained protein molecular weight standard. Lane 1, *E. coli* BL21 with empty vector pET-28a(+) reacted with polyclonal mouse anti-PEDV antibody. Lane 2, purified S1 protein reacted with polyclonal mouse anti-PEDV antibody. A prominent band with the expected size 42 kDa appeared after incubation

The gene of PEDV truncated S1 fragment (67–1134 nt) was amplified by RT-PCR (Fig. 1a). A 1068 bp PCR product was obtained and subcloned to prokaryotic expression vector pET-28a(+), and the inserted gene was sequenced to ensure the correctness of the reading frame. As shown by SDS-PAGE (Fig. 1c), the recombinant protein S1 was expressed in the form of inclusion body, resulting in a 6 × His-tag fusion protein whose molecular mass was approximately 42 kDa. Sonicated lysates from recombinant *E. coli* were harvested, and the precipitate was dissolved in 8 M urea and purified by affinity chromatography of Ni^2+^-NTA agarose. The immunoreactivity of S1 protein was examined by western blotting. An obvious band revealed that S1 protein was specifically bound by pig anti-PEDV polyclonal antibody (Fig. 1d).

### Optimization of S1 indirect ELISA

As expected with checkerboard titration, with concentrations of antigen and serum regularly decreasing, absorbance values of corresponding samples declined. The optimal dilution of coated antigen S1 protein was measured at 0.25 μg/well (2.5 μg/mL), and optimal serum sample dilution was 1:40 (Table 1). Furthermore, other reaction conditions of the developed ELISA were optimized. In brief, the optimum coating condition was 2 h at 37 °C. The best blocking solution was selected as 5% skimmed milk in PBS. The optimal reaction times for serum, secondary antibodies, and TMB solution were 45 min, 30 min and 15 min, respectively. Finally, the best working dilution of the HRP-goat anti-pig IgA was 1:10,000.

**Table 1:** Checkerboard titration of the recombinant protein S1

OD450 ratio (P/N)		Dilution of serum sample				
P/N		1:20	1:40	1:80	1:160	1:320
Concentrations of antigen (μg/mL)	10	1.233/0.185	0.980/0.100	0.730/0.082	0.551/0.052	0.401/0.041
		6.665	9.80	8.902	10.596	9.780
	7.5	1.118/0.150	0.942/0.092	0.631/0.075	0.459/0.047	0.309/0.029
		7.453	10.239	8.413	9.766	10.655
	5.0	1.102/0.141	0.854/0.077	0.591/0.067	0.388/0.042	0.228/0.022
		7.816	11.091	8.821	9.238	10.364
	2.5	1.032/0.120	0.832/0.073	0.547/0.063	0.335/0.040	0.195/0.019
		8.600	11.397	8.683	8.375	10.263
	1.0	0.927/0.112	0.753/0.071	0.473/0.057	0.316/0.037	0.183/0.017
		8.277	10.606	8.298	8.541	10.765
	0.5	0.818/0.103	0.714/0.068	0.447/0.055	0.292/0.033	0.171/0.016
		7.942	10.500	8.127	8.848	10.688

### Determination of the cut-off threshold value

To determine the cut-off value of the S1 indirect ELISA, 270 PEDV-seronegative samples, verified by both IFA and SN assays, were tested by this ELISA method. The average optical density of these negative serum samples (N) was calculated as 0.185, and the standard deviation (SD) of these samples was 0.0337. Consequently, the cut-off threshold value of S1 indirect ELISA was calculated to be 0.286, indicating that the sample OD_450_ ≥ 0.286 was identified as PEDV-seropositive and vice versa.

### S1 indirect ELISA validation

This developed S1 indirect ELISA was applied to 368 serum samples with varied PEDV antibody status (Table 2). In these samples, the S1 indirect ELISA detected 213 PEDV-positive samples, of which 206 tested PEDV-positive by IFA. On the other hand, of the remaining 155 samples that tested PEDV-seronegative by this S1 indirect ELISA, 150 of them were tested PEDV-negative by IFA. Hence, the sensitivity of S1 indirect ELISA was 96.71% among PEDV-seropositive individuals, and the specificity was 96.77% among PEDV-seronegative individuals using IFA as standard evaluation method. In summary, the overall coincidence rate of the S1 indirect ELISA to IFA was 96.74%.

**Table 2:** Comparison of the S1 indirect ELISA with the IFA

		IFA results		
		Positive	Negative	Total
S1 indirect ELISA results	Positive	206	7	213
	Negative	5	150	155
	Total	211	157	368
Data analysis	Sensitivity	96.71%		
	Specificity		96.77%	
	Coincidence rate			96.74%

### Cross-reactivity of S1 indirect ELISA

To test the cross-reactivity of this S1 indirect ELISA, other viruses known to cause swine diarrhea were examined. The average OD_450_ of positive serum samples for TGEV, PoRV, PKV, PBoV, PNoV, PCV2, PRRSV, ETEC, *Jerson Prand* of the small intestine, and *Clostridium Welchii* Type C were 0.193, 0.121, 0.098, 0.147, 0.150, 0.182, 0.178, 0.188, 0.145 and 0.124, respectively. The results showed that these serum samples were PEDV-seronegative and non-cross-reactive with this S1 indirect ELISA, indicating that the established ELISA was an effective method for detecting PEDV antibodies.

### The repeatability of S1 indirect ELISA

Intra-assay variability of the S1 indirect ELISA was assessed by testing 255 swine serum samples, each with 5 replicates. This analysis produced CVs ranging from 2.2–3.7%, with an average value of 2.8%. Inter-assay variability of four batches using identical samples produced CVs ranging from 2.6–4.5%, with an average value of 3.2%. The results demonstrated that this ELISA method yielded low levels of variation, and its repeatability was in the credible range.

### PEDV antibody detection of field serum samples

This S1 indirect ELISA method was used on 3304 swine serum samples of different PEDV exposure status collected from 37 farms (Table 3). In the 10 farms from which 1125 samples were collected during the PEDV outbreaks between 2011 and 2015, 1027 samples were tested PEDV antibody positive, the positive detection varied from 78 to 100% between 10 farms. In the 5 PEDV non-exposed farms from which 482 samples were collected between 2012 and 2015, 31 samples were tested PEDV antibody positive, the positive detection varied from 5.1 to 7.8% between 5 farms. Among PoRV or TGEV antibody positive serum samples, 16/149 anti-PoRV antibody positive sample and 27/195 anti-TGEV antibody positive samples were tested PEDV antibody positive. In the 16 farms from which 1353 samples with unknown PEDV exposure status were collected between 2011 and 2014, 491 samples were tested PEDV antibody positive, the positive detection varied from 15 to 47% between 16 farms.

**Table 3:** Detection rates of anti-PEDV antibodies in field serum samples

PEDV exposure	PoRV/TGEV exposure	No. samples	No. positive	Positive rate
Exposed	Non-exposed	1125	1027	91.29%
Non-exposed	Non-exposed	482	31	6.43%
Non-exposed	Exposed	149	16	10.74%
Non-exposed	Exposed	195	27	13.85%
Unknown	Unknown	1353	491	36.29%

Samples were collected from farms with known or unknown PEDV exposure as determined by real-time RT-PCR of fecal samples

### Comparation of IgA with IgG in the sera of PEDV immunized pigs

**Fig. 2:**
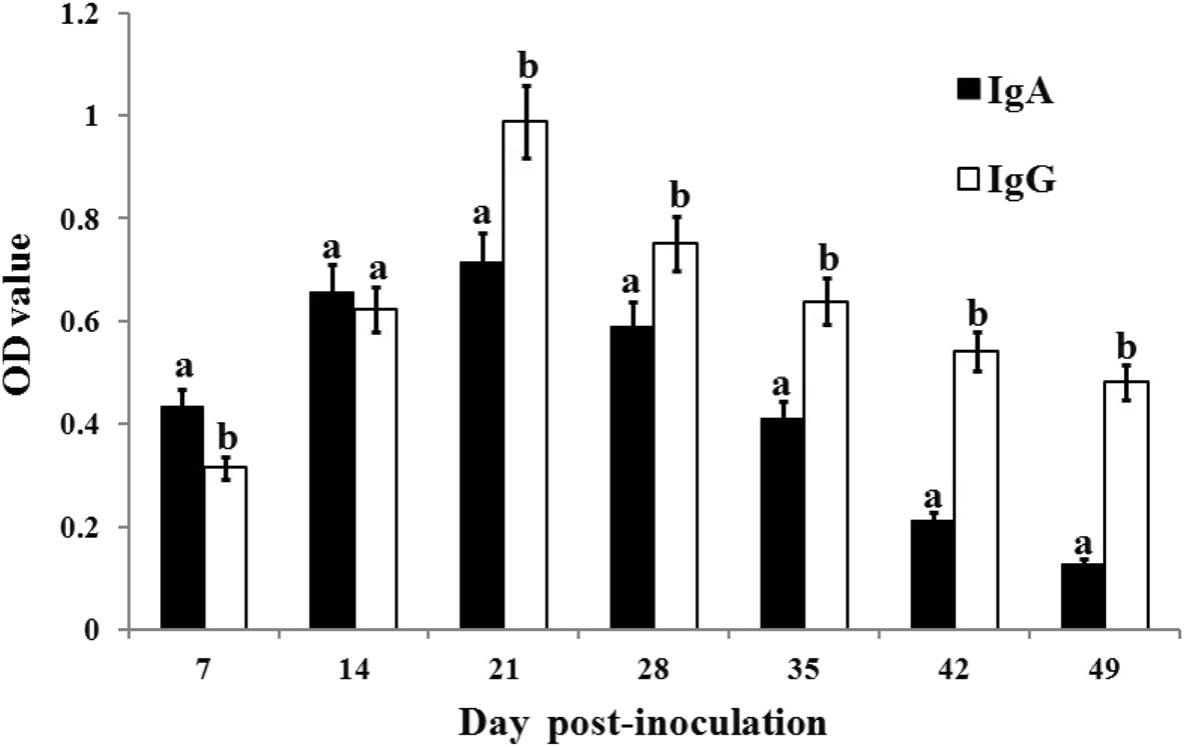
Determination of IgA and IgG in the sera of PEDV immunized pigs. The sera of PEDV immunized pigs were tested by this S1 indirect ELISA at 7, 14, 21, 28, 35, 42 and 49 day post-inoculation (dpi). Both the IgA and the IgG were positive at 7 dpi. The IgG could exist in the sera for longer time than IgA. Different letters (**a**, and **b**) indicate significant difference between the groups

This S1 indirect ELISA was applied to test the sera of PEDV immunized pigs at 7, 14, 21, 28, 35, 42 and 49 day post-inoculation (dpi). The results (Fig. 2) showed that, both the IgA and the IgG were positive at 7 dpi. At early infection stage (7 dpi), the IgA titer was significantly higher than the IgG titer; at 14 dpi the IgA titer was equivalent to the IgG titer; and after 21 dpi, the IgA titer was significantly lower than the IgG titer. The IgG could exist in the sera of infection recovered stage for more time than IgA.

## Discussion

Since December 2010, a large-scale outbreak of diarrhea has been observed in swine farms in China. Accumulated evidence indicates that this large-scale outbreak of diarrhea were caused by highly virulent PEDV variants [17, 18]. Serological assays can quickly detect large numbers of samples with both high sensitivity and specificity. Several indirect ELISA have been developed based on either whole PEDV preparations or recombinant viral proteins [19–21]. The S protein of PEDV has numerous epitopes and highly antigenic index regions that induce the production of neutralizing antibodies [10, 22, 23], and anti-S antibodies detected in PEDV-infected pigs persist longer than anti-N antibodies [11], thus, we choose S protein as the diagnostic antigen.

The recombinant S1 protein was applied to establish an indirect ELISA, and its reaction conditions were optimized. As PEDV is an enteric virus, it directly infects and damages enterocytes. Mucosal IgA, but not systemic IgG, plays a crucial role in protection [24, 25]. In this research, the titers of IgA in the serum were tested for serological evaluation and indirect diagnosis of PEDV infection. Of the 1125 serum samples which were PEDV exposed, the overall positive rate of the antibody is 91.29%, which were varied from 78 to 100% between 10 farms. Of the 482 serum samples which were PEDV non-exposed, the overall positive rate of the antibody is 6.43%, which were varied from 5.1 to 7.8% between 5 farms. The evaluated ELISA presented an overall substantial agreement on the PEDV infection status of the field swine serum samples.

The intra- and inter-assay variability tests proved that this ELISA method had good repeatability. When testing other positive serum related to swine viral pathogens, this established ELISA demonstrated no cross-reactivity to them. Further, the overall rate of coincidence of this ELISA was calculated at 96.74% compared with IFA, proving that the diagnostic sensitivity and specificity of this ELISA method were favorable.

Of the numerous pathogens which can cause swine viral diarrhea, PEDV, TGEV, and PoRV account for the largest proportion [26]. In the present study, 16/149 anti-PoRV antibody positive sample and 27/195 anti-TGEV antibody positive samples were tested PEDV antibody positive, which indicated there may be co-infection of PEDV and other virus.

## Conclusions

In conclusion, this established S1 indirect ELISA is capable of detecting serum antibodies against PEDV, and due to its high sensitivity and specificity, it could be applied for serological evaluation and indirect diagnosis of PEDV infection.

## Abbreviations

CV: Coefficient of variation
ELISA: Enzyme-linked immunosorbent assay
IFA: Indirect immunoinfluscent assay
PEDV: Porcine epidemic diarrhea virus (PEDV)
SD: Standard deviation
SN: Seroneutralization assay
TMB: Tetramethylbenzidine
